# Prevalence, incidence, and survival of pulmonary arterial hypertension: A systematic review for the global burden of disease 2020 study

**DOI:** 10.1002/pul2.12020

**Published:** 2022-01-18

**Authors:** Sophia Emmons‐Bell, Catherine Johnson, Alexandra Boon‐Dooley, Paul A. Corris, Peter J. Leary, Stuart Rich, Magdi Yacoub, Gregory A. Roth

**Affiliations:** ^1^ Institute for Health Metrics and Evaluation University of Washington Seattle Washington USA; ^2^ Translational and Clinical Research Institute, Faculty of Medical Sciences Newcastle University Newcastle upon Tyne UK; ^3^ Pulmonary Vascular Research Institute UK; ^4^ Division of Pulmonary, Critical Care, and Sleep Medicine University of Washington Seattle Washington USA; ^5^ Division of Cardiology Northwestern Memorial Hospital Chicago Illinois USA; ^6^ Aswan Heart Centre Aswan Egypt; ^7^ National Heart & Lung Institute Imperial College London London UK; ^8^ Harefield Heart Science Centre London UK; ^9^ Division of Cardiology, Department of Medicine University of Washington Seattle Washington USA

**Keywords:** population health, pulmonary arterial hypertension, survival

## Abstract

Pulmonary arterial hypertension (PAH) is characterized by increased resistance in the pulmonary arterioles as a result of remodeled blood vessels. We sought all available epidemiologic data on population‐based prevalence, incidence, and 1‐year survival of PAH as part of the Global Burden of Disease Study. We performed a systematic review searching Global Index Medicus (GIM) for keywords related to PAH between 1980 and 2021 and identified population‐representative sources of prevalence, incidence, and mortality for clinically diagnosed PAH. Of 6772 articles identified we found 65 with population‐level data: 17 for prevalence, 17 for incidence, and 58 reporting case fatality. Reported prevalence ranged from 0.37 cases/100,000 persons in a referral center of French children to 15 cases/100,000 persons in an Australian study. Reported incidence ranged from 0.008 cases/100,000 person‐years in Finland, to 1.4 cases/100,000 person‐years in a retrospective chart review at a clinic in Utah, United States. Reported 1‐year survival ranged from 67% to 99%. All studies with sex‐specific estimates of prevalence or incidence reported higher levels in females than males. Studies varied in their size, study design, diagnostic criteria, and sampling procedures. Reported PAH prevalence, incidence, and mortality varied by location and study. Prevalence ranged from 0.4 to 1.4 per 100,000 persons. Harmonization of methods for PAH registries would improve efforts at disease surveillance. Results of this search contribute to ongoing efforts to quantify the global burden of PAH.

## BACKGROUND

Pulmonary arterial hypertension (PAH) is a progressive and life‐threatening condition characterized by pulmonary vascular remodeling, increased resistance to blood flow through the pulmonary vasculature, and eventually right heart failure. An evaluation of current observed disease rates, with careful attention to the complex case definitions, is needed. The definition of PAH (World Health Organization [WHO] Group  1) has evolved over time and comprises a group of diseases including idiopathic, heritable, or drug‐induced PAH, previously called primary pulmonary hypertension. The updated WHO classification now includes the conditions associated with congenital heart disease, connective tissue disease, HIV, portal hypertension, and schistosomiasis. The diseases included in each subgroup have evolved over time. Systematic reviews of the epidemiologic literature for PAH must carefully account for the manner in which studies reported different definitions as classification systems evolved.

The Global Burden of Disease (GBD) study is a multinational effort to produce comparable, consistent measures of disease burden for national and subnational populations.^1^ The study leverages epidemiological relationships between prevalence, incidence, and mortality for hundreds of diseases to produce age‐, sex‐, and location‐specific estimates from 1990 onwards for use by scientists, policymakers, and health departments. While not explicitly estimated in prior iterations, the upcoming GBD 2020 study includes the first‐ever global and national estimates of PAH. To inform epidemiologic modeling efforts of PAH for GBD 2020, we attempted to identify all published population‐level data from 1990 to the present, including prevalence, incidence, and mortality, to produce high‐quality estimates of the burden of this fatal and understudied disease.

Previous systematic reviews of the literature have focused on non‐Group 1 pulmonary hypertension or specific subpopulations afflicted with PAH.^2^, ^3^, ^4^, ^5^, ^6^ A recent review focused on PAH prevalence and incidence from 2003 to 2020,^7^ but did not address case fatality. As the GBD estimates a full‐time series from 1990 to the present and includes all global locations, the inclusion of data before 2003 is essential, along with estimates of mortality with and without therapies. The purpose of this review was to rigorously focus on population representativeness in reviewing reported PAH prevalence, incidence, and mortality rates over the past three decades to identify inputs for future estimation of PAH for the GBD study.

## METHODS

### Case definition

Our literature review was designed to include all sources since 1990 while maintaining awareness of the evolving classification schema. Primary pulmonary hypertension was commonly reported before the Second World Symposium on Pulmonary Hypertension in 1998, which established five categories of pulmonary hypertension. After 1998, idiopathic, familial, and drug‐induced cases of pulmonary hypertension began to be reported as WHO Group 1 PAH.

Until 2019, hemodynamic criteria for PAH included a mean pulmonary arterial pressure (mPAP) of ≥25 mmHg and a pulmonary arterial capillary wedge pressure of <15 mmHg. In 2019, diagnostic criteria were revised to define PAH as an mPAP >20 mmHg (≥25 mmHg by previous guidelines), a wedge pressure ≤15 mmHg, and a pulmonary vascular resistance ≥3 Wood units.^8^ This revision is significant as estimates based on older hemodynamic criteria would represent a potential underestimation compared with the newer criteria.

Because echocardiography can lead to both under‐ and overdiagnosis of PAH in comparison to right heart catheterization (RHC), careful attention was paid to the manner in which echocardiography was used by each study protocol. Only six studies, with five reporting only survival and one reporting survival and prevalence, allowed the use of echocardiography to aid in the diagnosis of PAH for a subset of patients. Even for these studies, RHC was the primary mode of diagnosis and echocardiography was used with adherence to imaging guidelines and explicit exclusion for signs of left‐sided systolic or diastolic dysfunction. Only one study, performed in resource‐limited settings in Africa, was designed prospectively not to require RHC, however, assessments were performed by clinical specialists with a protocol intended to identify pulmonary hypertension and including formal evaluation of left‐heart function.^9^

The GBD study selects a case definition for each cause to ensure comparability between data sources and estimates and allows for adjustments to correct for any bias due to alternate definitions. The case definition for this literature review was based on physician diagnosis of WHO Group 1 PAH with supporting diagnostic evidence via RHC or, in only one case, echocardiography alone. PAH identified by the International Classification of Diseases (ICD) codes was included if the study authors had reviewed the patients' medical records and confirmed the diagnosis. We excluded studies restricted to subtypes within WHO Group 1 PAH, such as registries capturing only congenital, drug‐induced, or idiopathic PAH.

WHO Groups 2, 3, 4, and 5 pulmonary hypertension were excluded if recognized as such in the source manuscript.

### Data sources

Disease registries are one of the most important sources for understanding the demographics, clinical presentation, and outcomes of PAH. The first national registry of PAH was established in the United States in the early 1980s through the National Institutes of Health (NIH). In the coming decades, registries were established in the United States, France, United Kingdom, Spain, Germany, and Sweden.^10^ These registries vary in enrollment criteria, coverage, representativeness, number of centers, and type of participating centers. Other efforts to describe PAH have used health system or hospital‐derived administrative facility data using ICD codes.

### Search methodology

We conducted a structured review of the literature to identify all primary data sources reporting population‐representative estimates of prevalence, incidence, and case fatality of PAH. Our Preferred Reporting Items for Systematic Reviews and Meta‐Analyses (PRISMA) checklist is included in the appendix. We searched the Global Index Medicus (GIM), a library that indexes PubMed and international libraries including Latina American and Caribbean Health Sciences Literature and African Index Medicus, between 1980 and 2021 (GIM search string) and included all peer‐reviewed manuscripts. Since the original search in 2018, GIM has removed PubMed from its indexing; to account for this we searched PubMed independently for results from 2018 to 2021 and deduplicated the results in the final count. We reviewed results and added sources based on input from experts in the field of pulmonary vascular disease (S. R., P. L., P. C.).

We screened the titles and abstracts of all results. Full‐text review was performed for all studies with reported prevalence, incidence, or case fatality of PAH. We did not review studies reporting solely on Pulmonary Hypertension Groups 2–5. Studies were included based on the operant definition and classification at the time of the publication. As such, we report on a composite of several PAH definitions. Title/abstract screening and full‐text extraction were performed by two reviewers and arbitrated by a third. All included studies were reviewed by A. B. D., S. E. B, C. J., and G. R. Search strings are included in the appendix.

In full‐text review, we screened for representativeness, diagnostic criteria, and study type. To obtain representative population‐level estimates, we excluded studies that focused on one or only a few subpopulations. For example, we excluded studies in which the authors focused on only one race/ethnicity group among many within a country. Other studies were excluded if they focused on subgroups such as specific social groups or classes, or types of employees, data not representative of the location or geographic locations likely to lead to bias. Studies that sampled larger populations were included if the sampling method was representative of the population. We excluded papers without extractable data, such as descriptive reports of registries or when stratified in such a way as to obscure population‐level results.

We extracted all data at the most detailed age, sex, and location. Extractors were fluent in Spanish and English and used the support of colleagues or Google Translate to extract studies in other languages. We extracted the dates of the data, the location, the case definition, the study type, the percent of the study that was female, the mean age, and the mPAP (when given). When provided, we extracted confidence intervals or standard errors. When estimates of uncertainty were not published, we extracted cases and sample size and calculated standard errors using Wilson's score.

The review was not registered and study protocols were not published before this study. Data included in this review can be accessed through the Global Health Data Exchange (GHDX, http://ghdx.healthdata.org/).

## RESULTS

**Figure 1:**
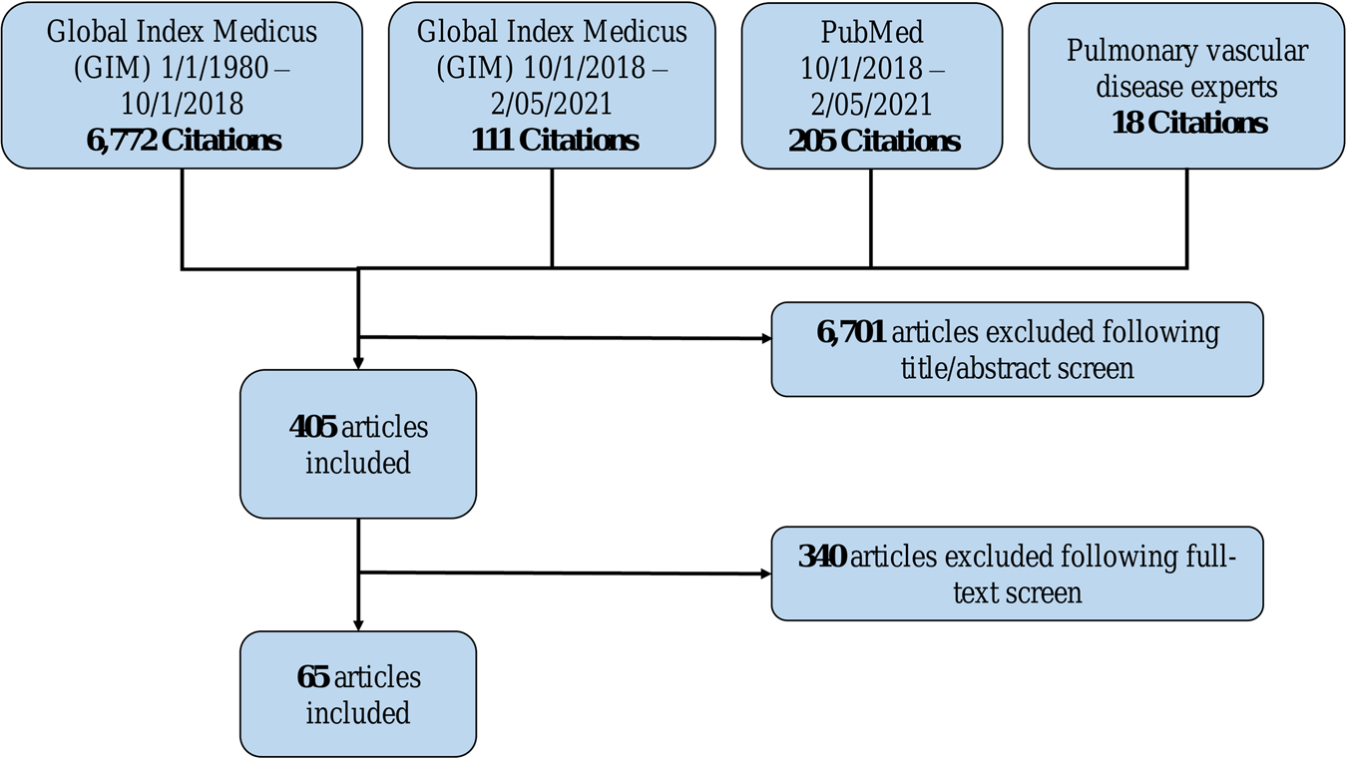
PRISMA diagram. Results of the systematic review, including number of hits from each data source, number of articles included after title/abstract review, and number of articles included

The search identified 6772 studies through February 5, 2021, of which 405 were selected for full‐text review and 65 were included in our analysis: 17 reporting prevalence estimates, 17 reporting incidence, and 58 reporting case‐fatality rate. Of these, 19 reported on more than one metric. All included studies were published between 1991 and 2021. See Figure 1 for the detailed flow of identified studies using a PRISMA‐style diagram. Thirty‐seven countries and 22 registries were represented (Table 1). Four studies reported only pediatric cases. All studies, except for one, had >50% female cases. All studies with sex‐specific estimates of prevalence or incidence reported higher levels in females than males. The breakdown of subtypes within WHO group 1 PAH reported by studies is included in the Supporting Information Table.

**Table 1:** 67 Studies reporting prevalence, incidence, and mortality of PAH identified in systematic review

First author, publication date	Location	Measure	Diagnosis type	Site type
Dubroff, Jason (2020)^11^	Utah	Prevalence	Right heart catheter	Single site
Dubroff, Jason (2020)^11^	Utah	Incidence	Right heart catheter	Single site
Khou, Victor, (2020)^12^	New Zealand and Australia	Crude mortality rate	Right heart catheter	Registry
Kopec, Grzegorz (2020)^13^	Poland	Prevalence	Right heart catheter	Registry
Kopec, Grzegorz (2020)^13^	Poland	Incidence	Right heart catheter	Registry
Kwiatkowska, Joanna (2020)^14^	Poland	Prevalence	Right heart catheter	Registry
Kwiatkowska, Joanna (2020)^14^	Poland	Incidence	Right heart catheter	Registry
Kwiatkowska, Joanna (2020)^14^	Poland	1‐year survival	Right heart catheter	Registry
Chazova, I. E. (2019)^15^	Russia	1‐year survival	Echocardiography + right heart catheter	Registry
Gruss, Ana (2019)^16^	Uruguay	Prevalence	Right heart catheter	Single site
Gruss, Ana (2019)^16^	Uruguay	Incidence	Right heart catheter	Single site
Gruss, Ana (2019)^16^	Uruguay	1‐year survival	Right heart catheter	Single site
Jang, Albert Youngwoo (2019)^17^	South Korea	1‐year survival	Right heart catheter	Multicenter
Gomes, A. (2018)^18^	Portugal	1‐year survival	Right heart catheter	Single site
Strange, Geoff (2018)^19^	Australia	1‐year survival	Right Heart Catheter	Multicenter
Austin, Christopher (2017)^20^	Florida	1‐year survival	Right heart catheter	Multicenter
Gall, Henning (2017)^21^	Germany	1‐year survival	Right heart catheter	Registry
Marques‐Alves, P. (2017)^22^	Portugal	1‐year survival	Right heart catheter	Registry
Quezada Loaiza, Carlos Andres (2017)^23^	Spain	1‐year survival	Right heart catheter	Single site
Tamura, Yuichi (2017)^24^	Japan	1‐year survival	Right heart catheter	Registry
Wang, Le‐Yung (2017)^25^	Taiwan	1‐year survival	Echocardiography + right heart catheter	Single site
Radegran, Goran (2016)^26^	Sweden	1‐year survival	Right heart catheter	Multicenter
Thienemann, Friedrich (2016)^27^	Four African Countries	6‐month survival	Echocardiography + right heart catheter if available	Registry
Vaid, Haris (2016)^28^	Canada	1‐year survival	electronic data capture	Multicenter
Chung, Wook‐Jin (2015)^29^	South Korea	1‐year survival	Echocardiography + right heart catheter	Multicenter
Farber, Harrison (2015)^30^	United States	1‐year survival	Right heart catheter	Registry
Grapsa, Julia (2015)^31^	United Kingdom	1‐year survival	Right heart catheter	Single site
Hoeper, Marius (2015)^32^	Germany	Prevalence	Right heart catheter	Registry
Hoeper, Marius (2015)^32^	Germany	Incidence	Right heart catheter	Registry
Idrees, Majdy (2015)^33^	Saudi Arabia	1‐year survival	Right heart catheter	Single site
Alves Jr, Jose Leonidas (2015)^34^	Brazil	1‐year survival	Right heart catheter	Single site
Mueller‐Mottet, Severine (2015)^35^	Switzerland	1‐year survival	Right heart catheter	Registry
Adachi, Shiro (2014)^36^	Japan	1‐year survival	Right heart catheter	Multicenter
Del Cerro Marin, M.J. (2014)^37^	Spain	1‐year survival	Electronic data capture	Multicenter
Del Cerro Marin, M. J. (2014)^37^	Spain	Incidence	electronic data capture	Multicenter
Del Cerro Marin, M. J. (2014)^37^	Spain	Prevalence	Electronic data capture	Multicenter
Jansa, Pavel (2014)^38^	Czech Republic	1‐year survival	Right heart catheter	Multicenter
Jansa, Pavel (2014)^38^	Czech Republic	Incidence	Right heart catheter	Multicenter
Jansa, Pavel (2014)^38^	Czech Republic	Prevalence	Right heart catheter	Multicenter
Korsholm, Kasper (2014)^39^	Denmark	1‐year survival	Right heart catheter	Registry
Talavera, Maria (2014)^40^	Argentina	1‐year survival	Right heart catheter	Single site
Zijlstra, Willemijn M. H. (2014)^41^	United States and Netherlands	1‐year survival	Right Heart Catheter	Multicenter
Baptista, Rui (2013)^42^	Portugal	1‐year survival	Right heart catheter	Registry
Baptista, Rui (2013)^42^	Portugal	Incidence	Right heart catheter	Registry
Ernande, Laura (2013)^43^	France, Belgium	1‐year survival	Right heart catheter	Multicenter
Frost, Adaani (2013)^44^	United States	1‐year survival	Right heart catheter	Registry
Roofthooft, Marcus T. R. (2013)^45^	Denmark	1‐year survival	Echocardiography + right heart catheter	Multicenter
Cogswell, Rebecca (2012)^46^	California	1‐year survival	Right heart catheter	Single site
Cracowski, Jean‐Luc (2012)^47^	France	1‐year survival	Right heart catheter	Multicenter
Escribano‐Subias, Pilar (2012)^48^	Spain	1‐year survival	Right heart catheter	Multicenter
Escribano‐Subias, Pilar (2012)^48^	Spain	Prevalence	Right heart catheter	Multicenter
Escribano‐Subias, Pilar (2012)^48^	Spain	Incidence	Right heart catheter	Multicenter
Ling, Yi (2012)^49^	United Kingdom and Ireland	Prevalence	Right heart catheter	Multicenter
Ling, Yi (2012)^49^	United Kingdom and Ireland	1‐year survival	Right heart catheter	Multicenter
Ling, Yi (2012)^49^	United Kingdom and Ireland	Incidence	Right heart catheter	Multicenter
Sakao, Seiichiro (2012)^50^	Japan	5‐year survival	Right heart catheter	Single site
Sakao, Seiichiro (2012)^50^	Japan	5‐year survival	Right heart catheter	Single site
Shapiro, Shelley (2012)^51^	United States	5‐year survival	Right heart catheter	Multicenter
Shimony, Avi (2012)^52^	Canada	1‐year survival	Right heart catheter	Single site
Strange, Geoff (2012)^53^	Australia	Prevalence	Right heart catheter	Multicenter
Wasywich, C. A. (2012)^54^	New Zealand	1‐year survival	Electronic data capture	Registry
Barst, Robyn (2011)^55^	United States	1‐year survival	Right Heart Catheter	Registry
Frost, Adaani (2011)^44^	United States	Incidence	Right heart catheter	Registry
Frost, Adaani (2011)^44^	United States	Prevalence	Right heart catheter	Registry
Hurdman, J. (2011)^56^	United Kingdom	1‐year survival	Electronic data capture	Single site
Kirson, Noam Y. (2011)^57^	United States	Prevalence	Electronic data capture	Multicenter
Kirson, Noam Y. (2011)^57^	United States	Prevalence	Electronic data capture	Multicenter
Low, A. J. (2011)^58^	Australia	1‐year survival	Right heart catheter	Multicenter
Sachdev, A. (2011)^59^	Minnesota	1‐year survival	Right heart catheter	Single site
Van Loon, Rosa Laura E. (2011)^60^	Netherlands	1‐year Survival	Electronic data capture	Registry
Fraisse, Alain (2010)^61^	France	1‐year survival	Echocardiography + right heart catheter	Multicenter
Fraisse, Alain (2010)^61^	France	Prevalence	Echocardiography + right heart catheter	Multicenter
Humbert, Marc (2010)^62^	France	1‐year survival	Right heart catheter	Registry
Carrington, Melinda (2008)^63^	Scotland	1‐year survival	Electronic data capture	Multicenter
Kim, Hyung Woo (2008)^64^	South Korea	5‐year survival	Right heart catheter	Single site
Tueller, Claudia (2008)^65^	Switzerland	Prevalence	Right heart catheter	Multicenter
Tueller, Claudia (2008)^65^	Switzerland	Incidence	Right heart catheter	Multicenter
Tueller, Claudia (2008)^65^	Switzerland	Incidence	Right heart catheter	Multicenter
Peacock, A. J. (2007)^66^	Scotland	Prevalence	Electronic data capture	Multicenter
Peacock, A. J. (2007)^66^	Scotland	Incidence	Electronic data capture	Multicenter
Thenappan, T. (2007)^67^	United States	1‐year survival	Right heart catheter	Multicenter
Humbert, Marc (2006)^68^	France	Prevalence	Right heart catheter	Registry
Humbert, Marc (2006)^68^	France	Incidence	Right heart catheter	Registry
Sankelo, Marja (2005)^69^	Finland	Prevalence	Electronic data capture	Multicenter
Sankelo, Marja (2005)^69^	Finland	Incidence	Electronic data capture	Multicenter
Appelbaum, Liat (2001)^70^	Israel	1‐year survival	Right heart catheter	Multicenter
Appelbaum, Liat (2001)^70^	Israel	Prevalence	Right heart catheter	Multicenter
Appelbaum, Liat (2001)^70^	Israel	Incidence	Right heart catheter	Multicenter
Okada, Osamu (1998)^71^	Japan	1‐year survival	Right heart catheter	Multicenter
Dantzker, David (1994)^72^	United States	1‐year survival	Right heart catheter	Registry
Rajasekhar, D. (1994)^73^	India	1‐year survival	Right heart catheter	Single site
Sandoval, Julio (1994)^74^	Mexico	1‐year survival	Right heart catheter	Single site
D'Alonzo, Gilbert (1991)^75^	United States	1‐year survival	Right heart catheter	Registry

*Note*: First author and publication date of 67 studies reporting prevalence, incidence, or survival of pulmonary arterial hypertension. Location refers to the location of patients or data collection; Measure refers to epidemiologic measures reported in the study; diagnosis type refers to diagnostic criteria used to identify pulmonary arterial hypertension; and site type refers to the type of study or site (e.g., registry, single site) used to identify patients.

**Figure 2:**
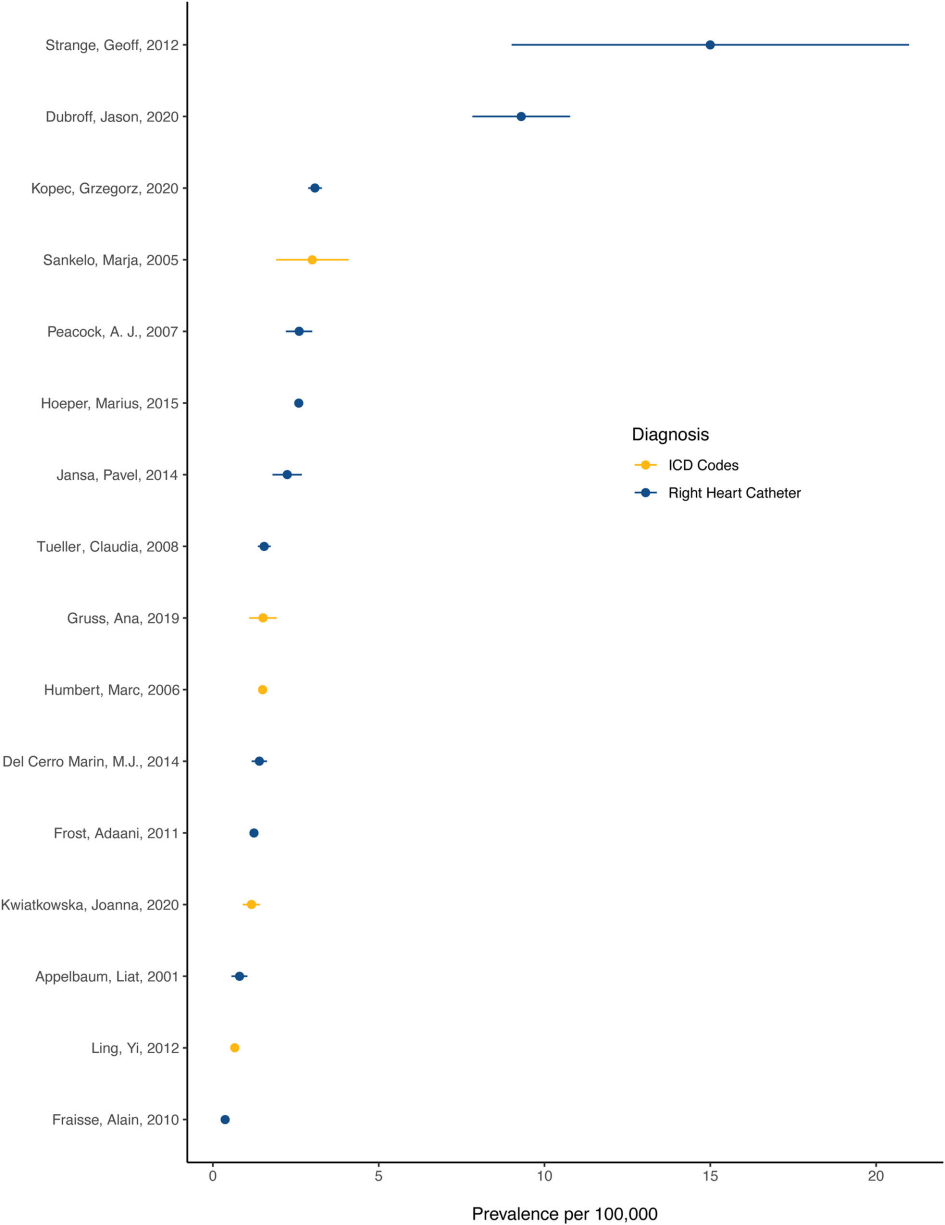
Reported prevalence of pulmonary arterial hypertension in 17 studies identified in systematic review *x*‐axis text: Prevalence per 100,000. Reported prevalence of pulmonary arterial hypertension per 100,000 as identified by echocardiography, right heart catheterization, echocardiography + right heart catheterization, or electronic data capture. Studies identified by first author and date of publication. Data represent reported prevalence and 95% confidence interval. When a confidence interval was not reported, we used cases and sample size to estimate a standard error using Wilson's score

Reported prevalence ranged from 0.37 cases/100,000, in a referral center of French children, to 15 cases/100,000, in an Australian study of a large database of echocardiograms (Figure 2). The simple mean of reported prevalence was 3.0 cases/100,000. Twelve studies diagnosed PAH through RHC, one through echocardiography with optional RHC, one through echocardiography and RHC, and three with ICD codes. Two studies reported on primary pulmonary hypertension, while the rest reported on PAH. Restricting to studies diagnosing PAH with RHC, the simple mean of reported prevalence was 3.7 cases/100,000. Restricting to studies diagnosing PAH with ICD codes, the mean of reported prevalence was 1.6 cases/100,000, and reported prevalence ranged from 0.66 cases/100,000 to 3 cases/100,000.

**Figure 3:**
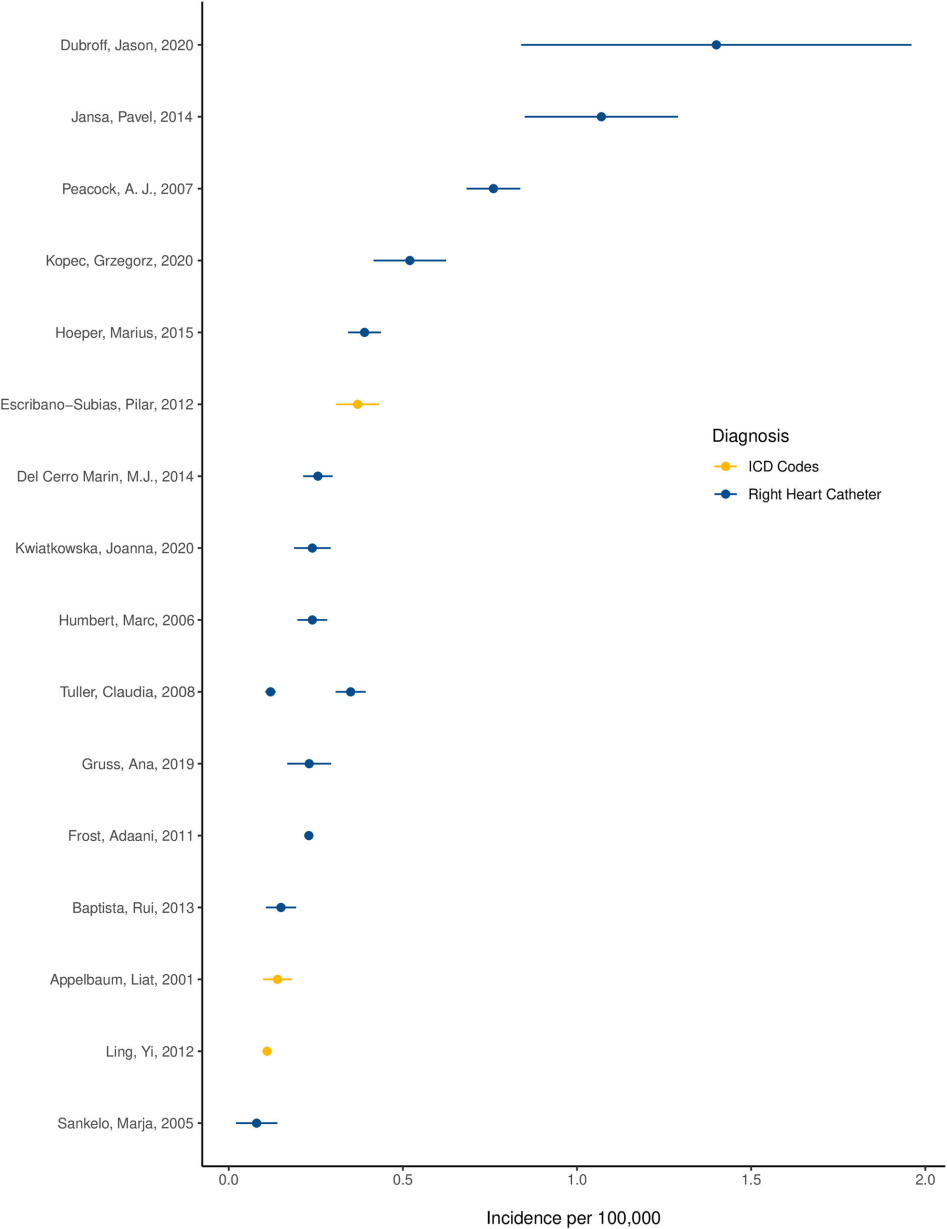
Reported incidence of pulmonary arterial hypertension in 17 studies identified in systemic review *x*‐axis text: Incidence per 100,000. Reported incidence of pulmonary arterial hypertension per 100,000 as identified by echocardiography or right heart catheterization. Studies identified by first author and date of publication. Data represent reported incidence and 95% confidence interval. When a confidence interval was not reported, we used cases and sample size to estimate a standard error using Wilson's score

Reported incidence ranged from 0.008 cases/100,000 person‐years, in a study of discharge records in Finland, to 1.4 cases/100,000 person‐years, in a retrospective chart review at a clinic in Utah, United States (Figure 3). The simple mean of reported incidence was 0.40 cases/100,000 person‐years. Fourteen studies diagnosed PAH through RHC, and three using electronic data capture. No papers reported on the incidence of primary pulmonary hypertension from the older classification schema. Restricting to studies diagnosing PAH with RHC, the simple mean of reported incidence was 0.43 cases/100,000 person‐years. Restricting to studies diagnosing PAH with ICD codes, the mean of reported incidence was 0.21 cases/100,000 person‐years, and reported incidence ranged from 0.11 cases/100,000 person‐years to 0.37 cases/100,000 person‐years.

**Figure 4:**
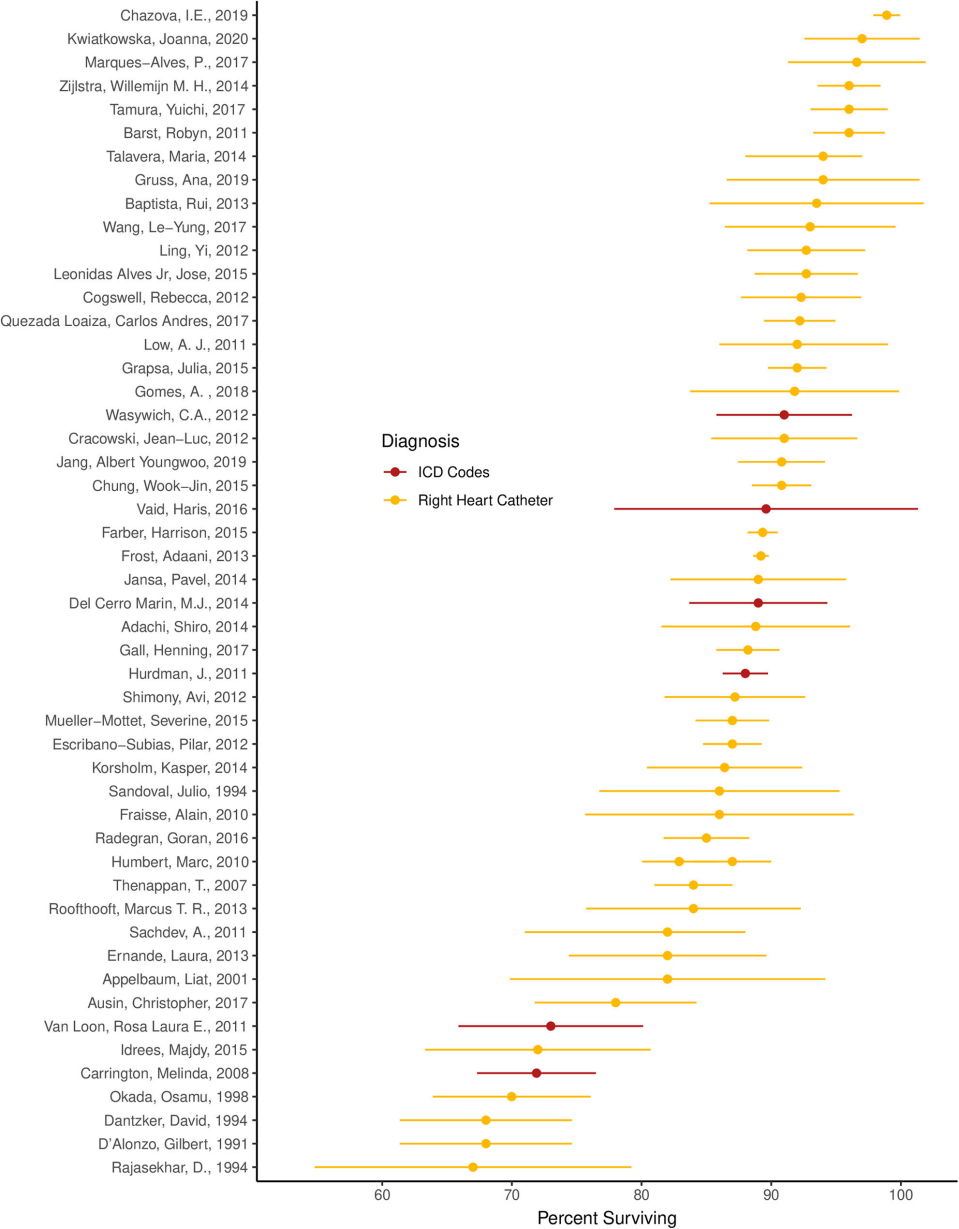
Reported 1‐year survival of pulmonary arterial hypertension in 58 studies identified in systematic review *x*‐axis text: Percent surviving 1 year. Reported 1‐year survival of pulmonary arterial hypertension per 100,000 as identified by echocardiography, right heart catheterization, echocardiography + right heart catheterization, or electronic data capture. Studies identified by first author and date of publication. Data represent reported incidence and 95% confidence interval. When a confidence interval was not reported, we used cases and sample size to estimate a standard error using Wilson's score

Reported 1‐year survival ranged from 67%, in a single‐center observational study in India in 1994, to 99%, in a 2019 study from the national PAH registry in Russia (Figure 4). The average reported 1‐year survival was 86%. Five studies reported 6‐month or 5‐year survival instead of 1‐year survival. Of the sources that reported on mortality, 44 used RHC to diagnose PAH, seven used echocardiography or a combination of echocardiography and RHC, and six with ICD codes. Five studies reported survival for primary pulmonary hypertension. Restricting to studies diagnosing PAH with RHC, the average reported 1‐year survival was 87%. Restricting to studies diagnosing PAH with ICD codes, the average reported 1‐year survival was 84% and reported 1‐year survival ranged from 72% to 91%.

Studies varied in size, case ascertainment strategy, diagnostic criteria, and design. Fifteen papers reported data from a single hospital or referral center, while others reported data from dozens of hospitals, multicenter registries, or national electronic medical systems. Single‐centers include specialty referral centers and nonreferral hospitals. Most studies estimated the catchment area of their center(s) to calculate the prevalence or incidence of PAH; some, like the Australian echocardiography study, performed community‐based sampling or used national insurance databases. Largely, studies with data before 1998 examined primary pulmonary hypertension, and studies with data after 1998 examined PAH. Many studies did not report the diagnostic criteria physicians used to diagnose PAH, and mPAP and echocardiography cutoffs varied slightly between studies. ICD codes used to diagnose PPH and PAH varied over time, due to the transition from ICD‐9 to ICD‐10.

## DISCUSSION

Estimates of prevalence, incidence, and mortality varied widely between studies with a 175‐fold difference in incidence between studies and a 40‐fold difference in prevalence. Although there may well be differences in local risk factors for PAH, such as methamphetamine use or congenital heart disease, there are no known risk factors of sufficient strength as to be likely to explain this level of variation.^1^, ^6^ As such, it is quite likely that a substantial amount of the variability in studies was related to the methodology used by individual studies including the approach to classifying PAH, centralized versus decentralized systems for tabulating PAH cases, and the generalized approach or resource limitations of the unique healthcare system in its ability and rigor in searching out PAH diagnoses. Harmonization of methods for PAH registries, especially diagnostic criteria, would improve efforts at comprehensive disease surveillance.

This search identified 65 studies reporting the prevalence, incidence, and 1‐year survival of PAH between 1991 and 2021 in 37 countries. These data will be used in the upcoming GBD study to estimate the global burden of PAH. The geographies represented in this review are largely in the high‐income world. There were very few studies in Africa, Eastern Europe, South America, and Asia, and papers from low‐ and middle‐income countries often reported survival, not prevalence or incidence. Challenges for the care of pulmonary hypertension in middle‐ and low‐income regions have been well‐described,^76^ but further surveillance is required to help understand the geographic variation in PAH incidence, prevalence, and survival.

These estimates are valuable both for resource allocation, but also as a benchmark for individual healthcare systems to understand if they are systematically missing the diagnosis of PAH or applying a label of PAH too liberally. Underdiagnosis has serious implications for individuals who are not recognized to have PAH. Conversely, over‐diagnosis has implications for society who may bear the significant costs for PAH treatment that typically runs to the hundreds of thousands of dollars per patient in highly resourced countries.^77^

The current effort found similar estimates of mean PAH prevalence and incidence as previous systematic reviews (incidence 0.4/100,000 person‐years vs. 0.58/100,000 person‐years, prevalence 3/100,000 vs. 5/100,000).^7^ The main contribution to differences between estimates was the inclusion of studies dating back to 1990, as well as those where PAH was diagnosed by echocardiography or electronic data capture. We include them to draw attention to the variation in criteria, case ascertainment, and case definition between studies.

Greater availability of PAH therapies has led to an improved prognosis as supported by a number of randomized controlled trials showing efficacy and improvement on current regimens including an impact on mortality.^78^ We observed higher mortality in studies reporting on data before 1998 than after 1998, although there were only four studies published before 1998. However, differences in case ascertainment, geographic representativeness, and diagnostic criteria make between‐study comparisons difficult. As such, we would recommend caution about over‐interpreting overall estimates of mortality trends.

Several registries were identified that did not meet our strict inclusion criteria due to exclusion of PH subtypes, however, provide important information on the epidemiology of some types of PAH.^79^, ^80^, ^81^ For example, 1‐year survival in idiopathic and familial PAH was found to be 68% in the Chinese Registry for PAH.^82^

Additionally, geographic comparison could provide insights into possible risk factors for PAH, as well as health system effectiveness, and location and comorbidity impacts on treatment. The inclusion of PAH in future iterations of the GBD study may provide the impetus for broader data collection efforts. More sophisticated proteomic and metabolomic approaches to classification may help guide future efforts to assess its true burden.^83^ The results of this search will be used to inform GBD estimation of PAH and help to quantify its global burden, thereby guiding global efforts to prioritize and treat this often‐overlooked condition.

## CONFLICT OF INTERESTS

Paul A. Corris serves on clinical trial committees for Acceleron, Bayer, and Johnson and Johnson.

## ETHICS STATEMENT

This study is part of the Global Burden of Diseases, Injuries, and Risk Factors study. The University of Washington IRB Committee approved the Global Burden of Diseases, Injuries, and Risk Factors study, STUDY00009060.

## AUTHOR CONTRIBUTIONS

Ms. Emmons‐Bell, Ms. Boon‐Dooley, Dr. Johnson, and Dr. Roth performed the systematic review. Ms. Emmons‐Bell performed the statistical analysis and prepared tables and figures. Ms. Emmons‐Bell, Dr. Johnson, and Dr. Roth drafted the manuscript. Dr. Corris, Dr. Leary, Dr. Rich, and Dr. Yacoub contributed critical analysis and revision of the manuscript.

## Supporting information

Supporting information.
