# Supplementary material for: Prevalence, incidence, and survival of pulmonary arterial hypertension: A systematic review for the global burden of disease 2020 study
Source: Pulm Circ. 2022 Jan 18;12(1):e12020. doi: 10.1002/pul2.12020 (PMC9052982; doi:10.1002/pul2.12020)
Supplement: Supplementary file 1 — Supporting information. [file PUL2-12-e12020-s001.docx]

# Supplementary Appendix

Emmons-Bell et al

## Contents

Search strings

PRISMA Checklist

Supplementary Table: Breakdown of subtypes of WHO Group 1 as reported in sources

## Search Strings

**GIM search string:** (tw:(“pulmonary arterial hypertension”) OR tw:(“pulmonary artery hypertension”) OR tw:(“primary pulmonary hypertension”) OR tw:(“group 1 pulmonary hypertension”) OR tw:(“group one pulmonary hypertension”)) AND (tw:(epidemiology) OR tw:(prevalent) OR tw:(prevalence) OR tw:(incident) OR tw:(incidence) OR tw:(“standardized mortality ratio”) OR tw:(“case fatality”) OR tw:(“relative risk of death”) OR tw:(“excess mortality”) OR tw:(“1-yr survival”)) AND NOT (tw:(rats) OR tw:(mice) OR tw:(dogs) OR tw:(apes) OR tw:(monkeys) OR tw:(chickens) OR tw:(pigs) OR tw:(sheep))

**PubMed search string:** (“pulmonary arterial hypertension”[Title] OR “pulmonary arterial hypertension”[Abstract] OR “pulmonary artery hypertension”[Title] OR “pulmonary artery hypertension”[Abstract] OR “primary pulmonary hypertension”[Title] OR “primary pulmonary hypertension”[Abstract] OR “group 1 pulmonary hypertension”[Title] OR “group 1 pulmonary hypertension”[Abstract] OR “group one pulmonary hypertension”[Title] OR “group one pulmonary hypertension”[Abstract]) AND (“epidemiology”[ Abstract] OR “prevalent cases”[ Abstract] OR “prevalence”[ Abstract] OR "incident cases"[ Abstract] OR “incidence”[ Abstract] OR “standardized mortality ratio”[ Abstract] OR “case fatality”[ Abstract] OR “relative risk of death”[ Abstract] OR “excess mortality”[ Abstract] OR “survival”[ Abstract]) NOT (animals[MeSH] NOT humans[MeSH])

## PRISMA Checklist

| **Section and Topic** | **Item #** | **Checklist item** | **Location where item is reported** |
| --- | --- | --- | --- |
| **TITLE** | | | |
| Title | 1 | Identify the report as a systematic review. | Title (1) |
| **ABSTRACT** | | | |
| Abstract | 2 | See the PRISMA 2020 for Abstracts checklist. | Abstract (2) |
| **INTRODUCTION** | | | |
| Rationale | 3 | Describe the rationale for the review in the context of existing knowledge. | Research in Context (3) |
| Objectives | 4 | Provide an explicit statement of the objective(s) or question(s) the review addresses. | Research in Context (3) |
| **METHODS** | | | |
| Eligibility criteria | 5 | Specify the inclusion and exclusion criteria for the review and how studies were grouped for the syntheses. | Search Methodology (5) |
| Information sources | 6 | Specify all databases, registers, websites, organisations, reference lists and other sources searched or consulted to identify studies. Specify the date when each source was last searched or consulted. | Search Methodology (5) |
| Search strategy | 7 | Present the full search strategies for all databases, registers and websites, including any filters and limits used. | Search String (Appendix) |
| Selection process | 8 | Specify the methods used to decide whether a study met the inclusion criteria of the review, including how many reviewers screened each record and each report retrieved, whether they worked independently, and if applicable, details of automation tools used in the process. | Search Methodology (5) |
| Data collection process | 9 | Specify the methods used to collect data from reports, including how many reviewers collected data from each report, whether they worked independently, any processes for obtaining or confirming data from study investigators, and if applicable, details of automation tools used in the process. | Search Methodology (5) |
| Data items | 10a | List and define all outcomes for which data were sought. Specify whether all results that were compatible with each outcome domain in each study were sought (e.g. for all measures, time points, analyses), and if not, the methods used to decide which results to collect. | Search Methodology (5) |
|  | 10b | List and define all other variables for which data were sought (e.g. participant and intervention characteristics, funding sources). Describe any assumptions made about any missing or unclear information. | Search Methodology (5) |
| Study risk of bias assessment | 11 | Specify the methods used to assess risk of bias in the included studies, including details of the tool(s) used, how many reviewers assessed each study and whether they worked independently, and if applicable, details of automation tools used in the process. | Search Methodology (5) |
| Effect measures | 12 | Specify for each outcome the effect measure(s) (e.g. risk ratio, mean difference) used in the synthesis or presentation of results. | Search Methodology (5) |
| Synthesis methods | 13a | Describe the processes used to decide which studies were eligible for each synthesis (e.g. tabulating the study intervention characteristics and comparing against the planned groups for each synthesis (item #5)). | Search Methodology (5) |
|  | 13b | Describe any methods required to prepare the data for presentation or synthesis, such as handling of missing summary statistics, or data conversions. | Search Methodology (5) |
|  | 13c | Describe any methods used to tabulate or visually display results of individual studies and syntheses. | Search Methodology (5) |
|  | 13d | Describe any methods used to synthesize results and provide a rationale for the choice(s). If meta-analysis was performed, describe the model(s), method(s) to identify the presence and extent of statistical heterogeneity, and software package(s) used. | Search Methodology (5) |
|  | 13e | Describe any methods used to explore possible causes of heterogeneity among study results (e.g. subgroup analysis, meta-regression). | N/A, no synthesis done |
|  | 13f | Describe any sensitivity analyses conducted to assess robustness of the synthesized results. | N/A, no synthesis done |
| Reporting bias assessment | 14 | Describe any methods used to assess risk of bias due to missing results in a synthesis (arising from reporting biases). | N/A, no synthesis done |
| Certainty assessment | 15 | Describe any methods used to assess certainty (or confidence) in the body of evidence for an outcome. | N/A, no synthesis done |
| **RESULTS** | | | |
| Study selection | 16a | Describe the results of the search and selection process, from the number of records identified in the search to the number of studies included in the review, ideally using a flow diagram. | Figure 1, PRISMA diagram |
|  | 16b | Cite studies that might appear to meet the inclusion criteria, but which were excluded, and explain why they were excluded. | Results (5) |
| Study characteristics | 17 | Cite each included study and present its characteristics. | Table 1 |
| Risk of bias in studies | 18 | Present assessments of risk of bias for each included study. | N/A, no synthesis done |
| Results of individual studies | 19 | For all outcomes, present, for each study: (a) summary statistics for each group (where appropriate) and (b) an effect estimate and its precision (e.g. confidence/credible interval), ideally using structured tables or plots. | Figure 2, 3, 4 |
| Results of syntheses | 20a | For each synthesis, briefly summarise the characteristics and risk of bias among contributing studies. | N/A, no synthesis done |
|  | 20b | Present results of all statistical syntheses conducted. If meta-analysis was done, present for each the summary estimate and its precision (e.g. confidence/credible interval) and measures of statistical heterogeneity. If comparing groups, describe the direction of the effect. | N/A, no synthesis done |
|  | 20c | Present results of all investigations of possible causes of heterogeneity among study results. | N/A, no synthesis done |
|  | 20d | Present results of all sensitivity analyses conducted to assess the robustness of the synthesized results. | N/A, no synthesis done |
| Reporting biases | 21 | Present assessments of risk of bias due to missing results (arising from reporting biases) for each synthesis assessed. | N/A, no synthesis done |
| Certainty of evidence | 22 | Present assessments of certainty (or confidence) in the body of evidence for each outcome assessed. | N/A, no synthesis done |
| **DISCUSSION** | | | |
| Discussion | 23a | Provide a general interpretation of the results in the context of other evidence. | Results (6) |
|  | 23b | Discuss any limitations of the evidence included in the review. | Discussion (6, 7) |
|  | 23c | Discuss any limitations of the review processes used. | Discussion (6, 7) |
|  | 23d | Discuss implications of the results for practice, policy, and future research. | Discussion (6, 7) |
| **OTHER INFORMATION** | | | |
| Registration and protocol | 24a | Provide registration information for the review, including register name and registration number, or state that the review was not registered. | Search Methodology (5) |
|  | 24b | Indicate where the review protocol can be accessed, or state that a protocol was not prepared. | Search Methodology (5) |
|  | 24c | Describe and explain any amendments to information provided at registration or in the protocol. | Search Methodology (5) |
| Support | 25 | Describe sources of financial or non-financial support for the review, and the role of the funders or sponsors in the review. | Page 1 |
| Competing interests | 26 | Declare any competing interests of review authors. | Page 1 |
| Availability of data, code and other materials | 27 | Report which of the following are publicly available and where they can be found: template data collection forms; data extracted from included studies; data used for all analyses; analytic code; any other materials used in the review. | Search Methodology (5) |

## Supplementary Table: Breakdown of subtypes of WHO Group 1 as reported in sources.

| **First Author, Publication Date** | **% Idiopathic** | **% Heritable** | **% CTD** | **% CHD** | **% Other** |
| --- | --- | --- | --- | --- | --- |
| Dubroff, Jason, 2020^19^ | 29.6 | NR | 34.4 | 6.5 | 29.3 |
| Khou, Victor, 2020^20^ | 39 | 3 | 36 | 11 | 11 |
| Kopec, Grzegorz, 2020^21^ | 45.8 | NR | 13.6 | 36.7 | 3.9 |
| Kwiatkowska, Joanna, 2020^22^ | 31.3 | NR | NR | 67.5 | 1.3 |
| Chazova, I.E., 2019^23^ | 41.5 | 0.4 | 19.5 | 36 | 2.7 |
| Gruss, Ana, 2019^24^ | 32.7 | NR | 25 | 34.6 | NR |
| Jang, Albert Youngwoo, 2019^25^ | NR | 23.2 | 49.8 | 25.4 | NR |
| Gomes, A., 2018^26^ | 29.1 | 3.6 | 20 | 36.4 | 10.9 |
| Strange, Geoff, 2018^27^ | 90.9 | 3.2 | NR | NR | 5.9 |
| Austin, Christopher, 2017^28^ | 43 | NR | 35 | 5 | 17 |
| Gall, Henning, 2017^29^ | 42.9 | NR | 21.2 | 13.3 | 22.6 |
| Marques-Alves, P., 2017^30^ | 12.3 | 1.5 | 24.6 | 47.7 | 13.8 |
| Quezada Loaiza, Carlos Andres, 2017^31^ | 43 | NR | 21 | 9 | 28 |
| Tamura, Yuichi, 2017^32^ | 55.6 | NR | 25.4 | 8.5 | 10.6 |
| Wang, Le-Yung, 2017^33^ | 20 | NR | 47.1 | 25.7 | 7.1 |
| Radegran, Goran, 2016^34^ | 49.7 | NR | 30.6 | 13.3 | 6.3 |
| Thienemann, Friedrich, 2016^35^ | NR | NR | NR | NR | NR |
| Vaid, Haris, 2016^36^ | NR | NR | NR | NR | NR |
| Chung, Wook-Jin, 2015^37^ | 23.2 | NR | 49.8 | 25.4 | 1.6 |
| Farber, Harrison, 2015^38^ | 46.6 | 3.1 | 24.3 | 11.6 | 13.2 |
| Grapsa, Julia, 2015^39^ | 36.4 | NR | 44.7 | 9 | 9.9 |
| Hoeper, Marius, 2015^40^ | 58.3 | NR | 16.4 | 18.9 | 6.4 |
| Idrees, Majdy, 2015^41^ | 51.4 | 3.7 | 15 | 27.1 | 2.8 |
| Alves Jr, Jose Leonidas, 2015^42^ | 28.7 | NR | 25.8 | NR | 30.4 |
| Mueller-Mottet, Severine, 2015^43^ | 60 | NR | 18 | 8 | 17 |
| Adachi, Shiro, 2014^44^ | 42 | NR | 34.6 | 19.7 | 3.7 |
| Del Cerro Marin, M.J., 2014^45^ | 21.8 | NR | 0.7 | 73.9 | 3.5 |
| Jansa, Pavel, 2014^46^ | 60.7 | 3.7 | 11 | 20.4 | 4.2 |
| Korsholm, Kasper, 2014^47^ | 32 | 0.8 | 23 | 35.8 | 8.8 |
| Talavera, Maria, 2014^48^ | 48.8 | 1.6 | 14.4 | 28 | 9 |
| Zijlstra, Willemijn M. H., 2014^49^ | 52 | NR | NR | 42 | 6 |
| Baptista, Rui, 2013^50^ | 37 | 2 | 26 | 22 | 13 |
| Ernande, Laura, 2013^51^ | 42.3 | NR | 26 | 12.5 | 19.2 |
| Frost, Adaani, 2013^52^ | NR | NR | NR | NR | NR |
| Roofthooft, Marcus T. R., 2013^53^ | 52.4 | NR | 3.7 | 37.8 | 6.1 |
| Cogswell, Rebecca, 2012^54^ | 55 | 1.4 | NR | NR | NR |
| Cracowski, Jean-Luc, 2012^55^ | 61.8 | 2.7 | 12.7 | 5.4 | 17.2 |
| Escribano-Subias, Pilar, 2012^56^ | 39.2 |  | 19.6 | 20.8 | 20.3 |
| Ling, Yi, 2012^57^ | 93 | 5 | NR | NR | 2 |
| Sakao, Seiichiro, 2012^58^ | 42.7 | NR | 37.9 | 7.8 | 11.7 |
| Shapiro, Shelley, 2012^59^ | 45.9 | 2.8 | 24 | 11.5 | 17 |
| Shimony, Avi, 2012^60^ | 44.8 | 1.3 | 40.3 | 5.8 | 7.8 |
| Strange, Geoff, 2012^61^ | 32 | NR | 16 | 52 | NR |
| Wasywich, C.A., 2012^62^ | 37 | 1 | 29 | 6 | 27 |
| Barst, Robyn, 2011^63^ | 56.5 | NR | 4.6 | 35.6 | 3.2 |
| Frost, Adaani, 2011^52^ | NR | NR | NR | NR | NR |
| Hurdman, J., 2011^64^ | 29.2 | NR | 31.3 | 33 | 6.5 |
| Kirson, Noam Y., 2011^65^ | NR | NR | NR | NR | NR |
| Low, A. J., 2011^66^ | 55.2 | NR | 24.1 | 13.8 | 6.9 |
| Sachdev, A., 2011^67^ | 43 | NR | 41 | NR | 16 |
| Van Loon, Rosa Laura E., 2011^68^ | 23.4 | NR | 1.9 | 72.1 | 2.6 |
| Fraisse, Alain, 2010^69^ | 60 | 10 | 4 | 24 | 2 |
| Humbert, Marc, 2010^70^ | 39.2 | 3.9 | 15.3 | 11.3 | 30.3 |
| Humbert, Marc, 2010^71^ | 80.5 | 6.3 | NR | NR | 13.2 |
| Carrington, Melinda, 2008^72^ | 47 |  | 30 | 23 |  |
| Kim, Hyung Woo, 2008^73^ | 16.9 | 3.1 | NR | 49.2 | 30.8 |
| Tueller, Claudia, 2008^74^ | 28 | NR | 29.2 | 4 | 38.8 |
| Peacock, A. J., 2007^75^ | 46.8 | NR | 29.7 | 23.5 | NR |
| Thenappan, T., 2007^76^ | 48 | NR | 30 | 11 | 11 |
| Humbert, Marc, 2006^77^ | 39.2 | 3.9 | 15.3 | 11.3 | 26.1 |
| Sankelo, Marja, 2005^78^ | NR | NR | NR | NR | NR |
| Appelbaum, Liat, 2001^79^ | NR | NR | NR | NR | NR |
| Okada, Osamu, 1998^80^ | NR | NR | NR | NR | NR |
| Dantzker, David, 1994^81^ | NR | NR | NR | NR | NR |
| Rajasekhar, D., 1994^82^ | NR | NR | NR | NR | NR |
| Sandoval, Julio, 1994^83^ | NR | NR | NR | NR | NR |
| D'Alonzo, Gilbert, 1991^84^ | NR | NR | NR | NR | NR |

### Supplementary table caption:

Percent of PAH patients classified as idiopathic PAH, familial or heritable PAH, connective tissue disease PAH, congenital heart disease PAH, or any other subtype. NR=Not Reported, both for single categories like heritable or entire papers. CTD=Connective Tissue Disease; CHD=Congenital Heart Disease; Other includes subtypes such as HIV, drug-induced, schistosomiasis; Idiopathic=Idiopathic and Heritable except where Heritable is reported.
